# Boosting Visible-Light Photocatalytic Activity of BiOCl Nanosheets via Synergetic Effect of Oxygen Vacancy Engineering and Graphene Quantum Dots-Sensitization

**DOI:** 10.3390/molecules29061362

**Published:** 2024-03-19

**Authors:** Zisheng Shi, Wei Chen, Yin Hu, Fen Zhang, Lingling Wang, Dan Zhou, Xuanye Chen, Sugang Meng

**Affiliations:** 1 School of Environment and Chemical Engineering, Nanchang Hangkong University, Nanchang 330063, China; victoryoflion@163.com (Z.S.); zhoudan@nchu.edu.cn (D.Z.); 2 Research Institute of Applied Chemistry, Jiangxi Academy of Sciences, Nanchang 330096, China; cathyzf@163.com (F.Z.); sunnyskywang@163.com (L.W.); xuanyechen11@163.com (X.C.); 3 Key Laboratory of Green and Precise Synthetic Chemistry and Applications, Ministry of Education, Huaibei Normal University, Huaibei 235000, China; mengsugang@126.com

**Keywords:** graphene quantum dots (GQDs), oxygen vacancy (V_O_), bismuth oxychloride (BiOCl), synergistic effect, photocatalytic degradation

## Abstract

In recent years, oxygen vacancy (V_O_) engineering has become a research hotspot in the field of photocatalysis. Herein, an efficient GQDs/BiOCl-V_O_ heterojunction photocatalyst was fabricated by loading graphene quantum dots (GQDs) onto BiOCl nanosheets containing oxygen vacancies. ESR and XPS characterizations confirmed the formation of oxygen vacancy. Combining experimental analysis and DFT calculations, it was found that oxygen vacancy promoted the chemical adsorption of O_2_, while GQDs accelerated electron transfer. Benefiting from the synergistic effect of oxygen vacancy, GQDs, and dye sensitization, the as-prepared GQDs/BiOCl-V_O_ sample exhibited improved efficiency for RhB degradation under visible-light irradiation. A 2 wt% GQDs/BiOCl-V_O_ composite effectively degraded 98% of RhB within 20 min. The main active species were proven to be hole (h^+^) and superoxide radical (·O_2_^−^) via ESR analysis and radical trapping experiments. This study provided new insights into the effective removal of organic pollutants from water by combining defect engineering and quantum dot doping techniques in heterojunction catalysts.

## 1. Introduction

Currently, the world is being confronted with significant challenges stemming from environmental degradation and energy scarcity. There is an urgent need for a solution to this major problem, to achieve sustainable development and enhance people’s living standards [1]. Semiconductor photocatalysis technology using solar energy has the capability to directly decompose environmental pollutants and perform thorough mineralization without causing secondary pollution [2]. As a consequence, conducting semiconductor photocatalysis research is of immense significance as it holds the potential to fundamentally solve environmental pollution problems [3,4]. Traditional wide-bandgap semiconductor photocatalysts, such as TiO_2_ and ZnO, primarily respond to short-wavelength ultraviolet (UV) light, which accounts for only ~5% of total solar energy, and photogenerated electrons and holes are prone to recombination, resulting in low solar light utilization and quantum efficiency [5]. To address these problems, the development of novel high-efficiency and wide-spectra-response photocatalysts is crucial.

BiOCl has indeed found widespread applications in the field of photocatalysis due to its numerous advantageous properties. Its low toxicity, stable chemical properties, wide distribution of constituent elements, and convenient regulation of the electronic band structure render it an attractive material for photocatalytic applications [6]. Furthermore, BiOCl possesses a unique anisotropic layered structure that enables the facile formation of two-dimensional (2D) nanosheets with high-exposure surfaces during crystal growth [7]. The efficiency of photocatalytic reactions is intimately related to the catalytic ability of their surface reaction sites, and defect engineering is the most effective method for constructing reaction active sites. Among the numerous recognized defects, oxygen vacancy is the most common and extensively studied anionic defect, with advantages such as easy construction, controllability, and significant enhancement in photocatalytic performance. To date, many studies have been reported on the application of oxygen vacancies in photocatalytic fields [8]. Benefiting from these properties, the construction of oxygen vacancies on BiOCl has been investigated in recent years. Zhang et al. successfully fabricated efficient BiOCl nanoplates with photoinduced oxygen vacancy for the photoreduction of CO_2_ [9]. Hao et al. synthesized abundant oxygen vacancy BiOCl via a facile solvothermal route toward oxygen evolution [10]. A strategy to promote photocatalytic O_2_ activation using sulfur-doped BiOCl with rich oxygen vacancies via modulation of the built-in electric field and manipulation of exciton was proposed by Huang et al. [11]. Qin et al. successfully synthesized BiOI/BiOCl heterojunctions with a reasonable concentration of interfacial oxygen vacancies using ultrasonication [12]. The photocatalyst of BiOI/BiOCl displayed remarkable degradation activity for 20 mg·mL^−1^ tetracycline hydrochloride, achieving an 84% degradation rate within 60 min. Yang et al. constructed dual vacancies on BiOCl for both oxygen and chlorine to improve O_2_ dissociation into monatomic reactive oxygen [13]. Qin et al. prepared oxygen vacancies on BiOCl via photoexcitation with UV light irradiation, thereby highlighting the significance of oxygen vacancies as the dynamic active sites in photocatalytic NO oxidation reactions [14]. In general, although the construction of oxygen vacancies have improved the light utilization and photocatalytic activity of BiOCl to a certain extent, common problems, such as narrow light absorption range and easy deactivation of the catalyst, have persisted.

GQDs, as flat zero-dimensional (0D) carbon materials, stand apart from other carbon materials. GQDs exhibit a unique quantum confinement effect, resulting in a narrower bandgap, excellent dispersibility, availability of numerous active sites (edges, functional groups, dopants, etc.), low toxicity, and environmental friendliness [15,16]. They are beneficial for chemical modification and are currently widely employed in the field of photocatalysis [17,18]. Moreover, GQDs can broaden the light response range of catalysts, serve as electron acceptors, facilitate electron transfer, and accelerate the separation of electrons and holes [19,20,21,22,23,24]. GQDs constitute a potential promoter for the catalyst composites. Therefore, the photocatalytic performance of catalysts could be enhanced by constructing GQD-based composite structures. Jia et al. discovered that reduced graphene quantum dots (r-GQDs) form a strong interfacial bond with TiO_2_ photocatalysts, enabling the efficient transfer of multiphoton-generated electrons from the r-GQDs to the TiO_2_ photocatalyst [19]. The application of r-GQDs/TiO_2_ composites with high electron transfer capability to overall water splitting (H_2_: 60.4µmol·g^−1^·L^−1^ and O_2_: 60.4 µmol·g^−1^·L^−1^) and CO_2_ reduction under infrared light has been found to be noteworthy. Rama Shanker Sahu et al. prepared a CN@GQDs metal-free composite via a two-step process that involved high-temperature calcination followed by hydrothermal treatment [21]. Cui et al. prepared GQDs/BWO composites through combining CA pyrolysis and hydrothermal methods. The introduction of GQDs to the composite increased the specific surface area, providing more reactive sites and enhanced light absorption to promote carrier separation and establish photogenerated electron channels [24]. The modification of the GQDs helped to increase the visible-light absorption efficiency, reduce the band gap, and enhance hydrophilicity.

The synergistic promotion of the separation and transfer of photogenerated charges through the construction of heterojunctions and the formation of oxygen vacancy defects provide considerable potential in photocatalysis. The formation of heterojunctions in the region of oxygen vacancy defects helped solve the stability problem of oxygen vacancy defect structures, ensuring optimal charge transfer efficiency and accuracy. GQDs, as derivatives of graphene, have excellent beneficial properties comparable to metals. Hence, GQDs can be claimed to be an excellent choice for constructing heterojunctions. The synergetic effect of GQDs and oxygen vacancies may achieve unexpected photocatalytic performance. In addition, dyes are used as both degradation models and photosensitization compounds in photocatalysis systems. Dye sensitization involves the enhancement of light absorption into the visible region via the binding of functional groups of dyes (–COOH, –OH, etc.) to the surface of the semiconductor.

Therefore, we adopted a control strategy that mainly focused on constructing heterojunctions and forming oxygen vacancy defects, and constructed GQDs/BiOCl-V_O_ heterojunction photocatalysts for the degradation of Rhodamine B (RhB) under visible-light. The introduction of both oxygen vacancies and GQDs enhanced the photocatalytic activity of the BiOCl nanosheets. We systematically studied the synergistic mechanism between oxygen vacancies and GQDs, and the mechanism for enhancing photocatalytic performance. Through a series of characterizations and DFT theoretical calculations, the main active species in the photocatalytic process were revealed, interface interaction and the mechanism of photogenerated charge separation and transport were explored, and the mechanism of photocatalytic degradation was proposed. This study integrated oxygen vacancy defects engineering with quantum dot doping technology, providing new insights into the effective elimination of organic pollutants in water by finely regulating space charge transfer and separation.

## 2. Results and Discussion

### 2.1. XRD, DRS, and PDOS Analysis

**Figure 1:**
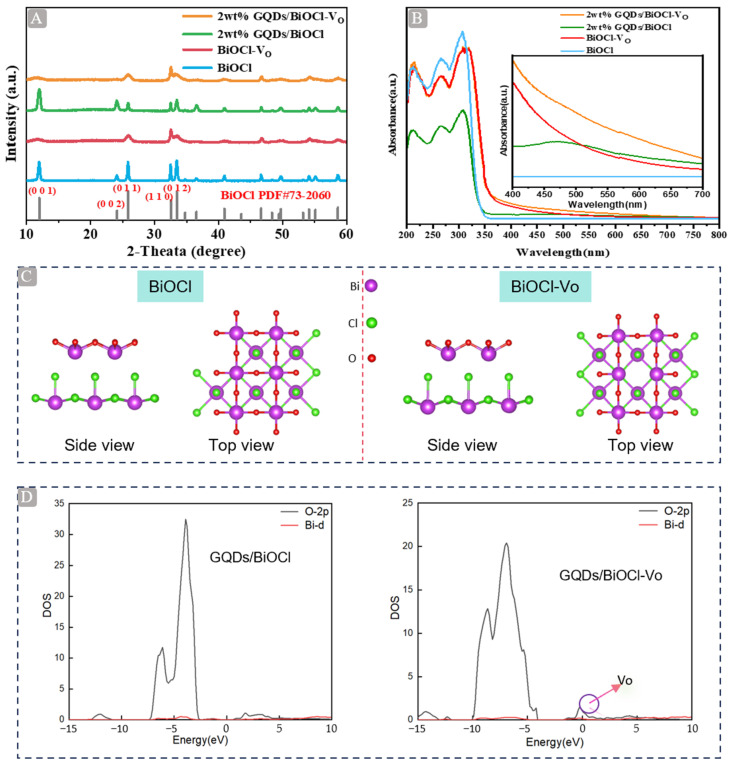
(**A**) XRD pattern of BiOCl, BiOCl-V_O_, 2 wt% GQDs/BiOCl, and 2 wt% GQDs/BiOCl-V_O_. (**B**) UV-vis DRS pattern of BiOCl, BiOCl-V_O_, 2 wt% GQDs/BiOCl, and 2 wt% GQDs/BiOCl-V_O_ (inset image: enlarged UV-vis DRS pattern). (**C**) Crystal structure of BiOCl and BiOCl-V_O_. (**D**) Partial density of states (PDOS) of GQDs/BiOCl and GQDs/BiOCl-V_O_. Note: the colored balls represent different atoms: red, oxygen (O); green, chlorine (Cl); purple, bismuth (Bi).

The phase purity and crystallinity of the samples were confirmed through X-ray diffraction (XRD) analysis. Figure 1A shows the XRD patterns of BiOCl, BiOCl-V_O_, 2 wt% GQDs/BiOCl, and 2 wt% GQDs/BiOCl-V_O_. The characteristic peaks located at 12.03°, 24.2°, 25.93°, 32.58°, and 33.55° were assigned to the (001), (002), (012), (110), and (112) facets of the tetragonal BiOCl (JCPDS no. 73–2060), respectively [25]. No diffraction peaks for GQDs were found in the composites, possibly due to the small size and the trace amount, as well as the uniform dispersion on the BiOCl-V_O_, agreeing with similar reports for other GQDs-based composites [19,20]. Notably, the diffraction peak widths at half-maximum of BiOCl-V_O_ were larger than those of BiOCl, which agreed well with previous results, and could be associated with the formation of oxygen vacancies and residual surface stress arising from GQDs on BiOCl-V_O_ [10]. As shown in Figure S1A, the introduction of GQDs did not change the crystal structure of BiOCl-V_O_. Comparing BiOCl with BiOCl-V_O_ in Figure S1B, the diffraction peaks of the (002) facet disappeared in the XRD pattern of BiOCl-V_O_, which confirmed the smaller size and thinner structure of BiOCl-V_O_ with many oxygen vacancies [26]. The microstructural features of as-prepared samples obtained from XRD analysis are shown in Table S1. The introduction of GQDs and oxygen vacancies into BiOCl had little effect on most cell parameters. With the introduction of oxygen vacancies and GQDs, the crystallite size showed a significant reduction. All as-prepared samples exhibited a tetragonal structure; thus, a = b, and α = β = 90°. 

The optical properties of 2 wt% GQDs/BiOCl-V_O_, 2 wt% GQDs/BiOCl, BiOCl-V_O_, and BiOCl were explored via UV-vis diffuse reflectance spectrometry (DRS). It is well known that both oxygen vacancies and GQDs can greatly affect the optical properties of BiOCl. As shown in Figure 1B, BiOCl could only absorb UV light [27]. With the introduction of oxygen vacancies and GQDs, the light-absorbing band edge emergence slightly red-shifted. Concurrently, the light-absorbing band edge exhibited exponentially decaying visible-light trailing absorption that covered the region of 400–700 nm, as shown in the inset of Figure 1B [28]. The 2 wt% GQDs/BiOCl-V_O_ sample displayed the highest light absorption compared with the remaining photocatalysts. There are two potential reasons for enhanced visible light harvesting. As a photosensitive material, GQDs have the ability to enhance the capture of visible light [24]. Meanwhile, the introduction of oxygen vacancies transformed the color of the catalyst from white to gray (Figure S1C), thereby enhancing light absorption [9]. Figure S1D shows the band energies of BiOCl and BiOCl-V_O_, which were calculated using the formula ${\left(\alpha hv\right)}^{1/n}=A(hv-E_{g})$, where *α*, *h*, *ν*, *E_g_*, and *A* are the absorption coefficient, Planck constant, light frequency, band gap, and a constant, respectively. Because the band gap of BiOCl was an indirect semiconductor, the value of *n* was 2 [11]. The band gaps of BiOCl and BiOCl-V_O_ were 3.67 and 3.51 eV, respectively, indicating that the introduction of oxygen vacancies could alter the band energy.

The crystal structure and partial density of states (PDOS) were constructed. Figure S2 shows that the surface HOMO and LUMO of GQDs/BiOCl and GQDs/BiOCl-V_O_ samples were mainly composed of O 2p electrons. For the GQDs/BiOCl-V_O_ sample, the charge transfer between the GQDs and BiOCl was more obvious, facilitating the separation of photogenerated electrons. Meanwhile, the crystal structure models of BiOCl and BiOCl-V_O_, and the PDOS of GQDs/BiOCl and GQDs/BiOCl-V_O_ were calculated, as shown in Figure 1C,D. The conduction band edge of GQDs/BiOCl-V_O_ became more negative than that of GQDs/BiOCl, exhibiting enhanced reducing ability. Oxygen vacancy introduced an impurity level into the band gap of BiOCl, which reduced its band gap and increased visible-light absorption, consistent with the DRS results [10].

### 2.2. TEM and BET Analysis

**Figure 2:**
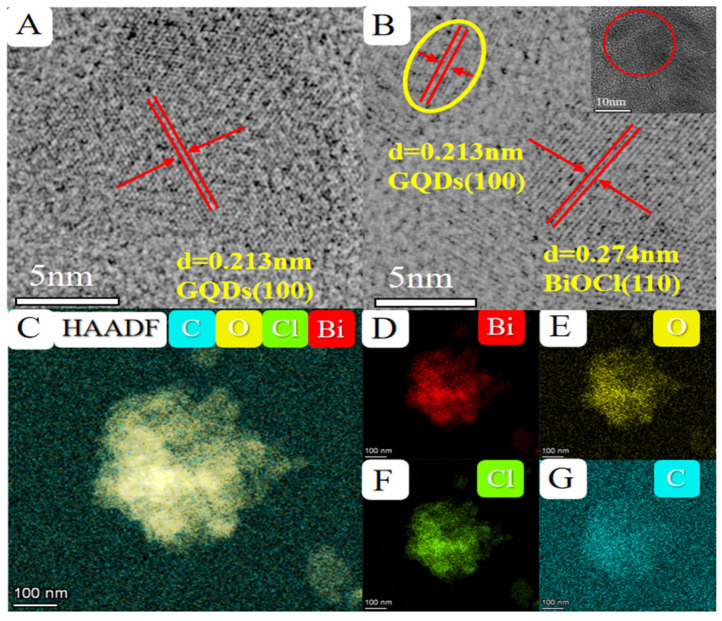
(**A**) Enlarged HRTEM of GQDs. (**B**) Enlarged HRTEM of the GQDs/BiOCl-V_O_. (**C**–**G**) The elemental mapping of GQDs/BiOCl-V_O_.

The objective was to explore the microstructure and morphology information of the GQDs/BiOCl-V_O_ sample. Characterizations were performed using transmission electron microscopy (TEM), high-resolution transmission electron microscopy (HRTEM), and elemental mapping. GQDs/BiOCl-V_O_ were prepared via a simple hydrothermal treatment, as shown in Scheme S1. The HRTEM of GQDs (Figure S3A) indicated that GQDs of 5–10 nm size dispersed in aqueous solution. Figure 2A shows clear interphase lattice fringes at 0.213 nm, corresponding to where the (100) plane of GQDs was found [24]. Figure 2B reveals that the lattice spacing of 0.274 nm and 0.213 nm matched the (110) plane of BiOCl and the (100) plane of GQDs, demonstrating the successful decoration of the GQDs on BiOCl-V_O_ nanosheets [29]. This result aligned with the aforementioned XRD findings. The formation of oxygen vacancies may be attributed to thin nanosheets with poor crystallinity [26]. Figure S3B shows the TEM of the GQDs/BiOCl-V_O_, which clearly demonstrates the structure of the sample as a sheet of 40–80 nm in length, echoing the scheme of preparation. Moreover, white spots on the surface of BiOCl nanosheet surfaces might be caused by oxygen lattice defects [12]. In order to indicate GQDs successfully modified on BiOCl-V_O_, elemental mapping was conducted. In the elemental mapping (Figure 2C−G), it was observed that elements of Bi, O, and Cl were seen uniformly dispersed in GQDs/BiOCl-V_O_, and a small amount of C from GQDs was uniformly distributed on the GQDs/BiOCl-V_O_ sample.

In addition, Figure S4A shows the nitrogen adsorption-desorption isotherm plots for as-prepared samples. Based on IUPAC classification, the isotherms of all as-prepared samples are type IV isotherms with type H_2_ hysteresis loops, suggesting that the catalyst has a mesoporous structure [30]. The details are shown in Table S2. The decrease in BET surface area and average pore diameter was mainly influenced by oxygen vacancies, while the influence of GQDs was not significant. The results show that the BET surface area and average pore diameter were not the sole determining factors in enhancing the catalytic performance of 2 wt% GQDs/BiOCl-V_O_.

### 2.3. IR, XPS, and ESR Analysis

**Figure 3:**
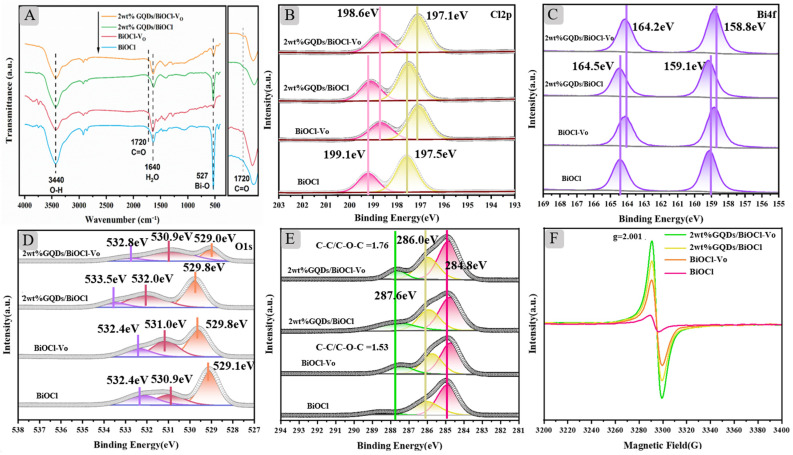
(**A**) FTIR spectra of different samples. High-resolution XPS spectra: (**B**) Cl 2p; (**C**) Bi 4f; (**D**) O 1s; (**E**) C 1s. (**F**) ESR spectra of BiOCl, BiOCl-V_O_, 2 wt% GQDs/BiOCl, and 2 wt% GQDs/BiOCl-V_O_.

To verify the effective introduction of GQDs onto the surface of BiOCl, Fourier transform infrared spectroscopy (FTIR) was recorded for the prepared samples (Figure 3A). Due to the low loading amount, the weak characteristic peaks observed at 1720 cm^−1^ were attributed to the stretching vibration of the C=O bond of GQDs, as shown in the enlarged image on the right [31]. It demonstrated that GQDs were loaded onto the BiOCl support. The characteristic peaks observed at 3440 cm^−1^ and 527 cm^−1^ were attributed to the stretching vibration peaks of O–H and Bi–O bond, respectively. The peak at 1640 cm^−1^ arose from the O–H stretching vibration of adsorbed water on the catalyst surface.

To explore the chemical properties of the prepared samples, the surface chemical composition and valence state of the composites were carefully examined using X-ray photoelectron spectroscopy (XPS). All XPS spectra were corrected relative to C 1s at 284.8 eV. Figure S4B depicts the XPS full spectrum of the as-prepared samples, representing all the elements present in the respective sample. For intuitive observation, Table S3 lists the relative peak areas of the components in the element. Figure 3B shows that the Cl 2p spectrum was accurately fitted with two peaks at 196.9–197.5 eV and 198.6–199.1 eV, corresponding to the Cl 2p_3/2_ and Cl 2p_1/2_, respectively [32]. Afterward, the Cl 2p XPS spectra shifted toward lower binding energy due to the introduction of oxygen vacancies, resulting in an excess of electrons for Cl [33]. In the high-resolution spectrum of Bi displayed in Figure 3C, the peaks were situated at 159.1 and 164.4 eV, indicating the presence of Bi^3+^ in all samples [34]. In addition, when compared with BiOCl, the Bi 4f_5/2_ and Bi 4f_7/2_ peaks of BiOCl-V_O_ shifted toward lower binding energy, suggesting the reduction of Bi^3+^ to lower oxidation states [34]. The peaks in the high-resolution XPS spectra of O 1s (Figure 3D) were located at 529.2, 530.6 and 532.3 eV, corresponding to the crystal lattice, oxygen vacancies, and surface absorbed oxygen in the samples, respectively [35]. Notably, the content of oxygen vacancy was listed in descending order of abundance as 2 wt% GQDs/BiOCl-V_O_, 2 wt% GQDs/BiOCl, BiOCl-V_O_, and BiOCl. In the high-resolution XPS spectra of C in Figure 3E, the peaks of different samples located at 284.8, 286.0, and 287.6 eV were attributed to C–C, C–O–C, and O–C=O, respectively [33]. The C 1s spectrum of BiOCl only showed C–C and C–O resulting from adventitious carbon. However, the C 1s spectrum of 2 wt% GQDs/BiOCl showed an additional peak compared with BiOCl, which was attributed to the presence of GQDs [20]. The intensity ratio of C–C/C–O–C was 1.76 in the composite of 2 wt% GQDs/BiOCl-V_O_, which was higher than that of BiOCl, with 1.53. This could be C–O–C attributed to the C–C skeletons in the GQDs [33]. This outcome vindicated the successful integration of GQDs into BiOCl-V_O_.

To confirm the presence of oxygen vacancies in the samples, electron spin-resonance spectroscopy (ESR) of different samples was obtained, as shown in Figure 3F. It was not difficult to observe the introduction of oxygen vacancies with high intensity. The appearance of the characteristic signal was at 2.001, which was identified as coming from an electron trapped in the oxygen vacancy [36]. The introduction of PVP would increase the content of oxygen vacancies, and the introduction of GQDs would improve the intensity of oxygen vacancies, consistent with the results of XPS.

### 2.4. Degradation Performance and Stability

At the early stages of the experiment, we delved into the optimal temperature and duration for the preparation of BiOCl-V_O_. Figure S5A,B illustrate the influence of hydrothermal temperature and time on the photocatalytic activity of BiOCl-V_O_. Samples synthesized at different temperatures and time show varying activities, while the catalyst prepared at 160 °C for 3 h exhibited the optimized activity.

**Figure 4:**
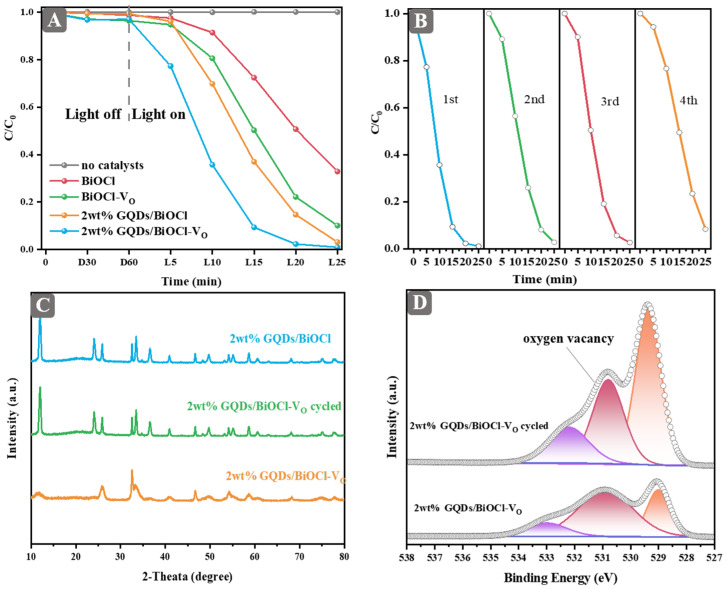
(**A**) Comparison of RhB degradation ratio of no catalysts, BiOCl, BiOCl-V_O_, 2 wt% GQDs/BiOCl, and 2 wt% GQDs/BiOCl-V_O_ under visible-light irradiation. (**B**) Stability test of GQDs/BiOCl-V_O_ using four-run recycling experiments under visible-light irradiation. (**C**) XRD patterns of 2 wt% GQDs/BiOCl, 2 wt% GQDs/BiOCl-V_O,_ and 2 wt% GQDs/BiOCl-V_O_ cycled. (**D**) High-resolution XPS spectra of O1s of 2 wt% GQDs/BiOCl-V_O_ and 2 wt% GQDs/BiOCl-V_O_ cycled.

The photocatalytic degradation of RhB was performed under visible-light irradiation. The photocatalytic performance of BiOCl and BiOCl-V_O_ decorated with varying contents of GQDs were tested. In the GQDs/BiOCl system (Figure S5C), the activity of BiOCl was enhanced as the dosage of GQDs increased. In another system (Figure S5D), the performance of BiOCl-V_O_ was enhanced by the introduction of GQDs. The 2 wt% GQDs/BiOCl-V_O_ exhibited the best performance in this system. As shown in Figure 4A, the increased catalytic activity of BiOCl and BiOCl-V_O_ was closely associated with the formation of oxygen vacancies. Moreover, 2 wt% GQDs/BiOCl-V_O_ demonstrated significantly higher photocatalytic activity for RhB degradation than BiOCl, BiOCl-V_O_, and 2 wt% GQDs/BiOCl. After 20 min, the photodegradation efficiency for RhB was 98% for 2 wt% GQDs/BiOCl-V_O_, 87% for 2 wt% GQDs/BiOCl, 78% for BiOCl-V_O_, and 50% for BiOCl. RhB degradation facilitated by these catalysts was also conducted under UV light activation. As shown in Figure S6A, RhB was stable under UV light. It showed lower performance than the reaction under visible light, presumably due to the absence of a RhB-sensitization effect. However, the synergistic effect of oxygen vacancy and GQDs was still obvious for improving the photocatalytic performance. In addition, the degradation performance of the antibiotic norfloxacin was tested (Figure S6B). The results showed that 2 wt% GQDs/BiOCl-V_O_ presented the best photocatalytic degradation performance. Norfloxacin can be not degraded without the catalyst under visible-light irradiation. The weak activity over bare BiOCl was probably caused by a small amount of oxygen vacancies, confirmed by ESR results (Figure 3F). These results confirmed the positive synergistic effect of oxygen vacancy and GQDs on the enhanced photocatalytic performance of BiOCl.

To evaluate the potential application and commercial value of the photocatalysts, cycling experiments were carried out under identical conditions. After each recycling run, the sample was washed with water and ethanol, and dried overnight at 60 °C before being reused. The degradation efficiency of 2 wt% GQDs/BiOCl-V_O_ decreased after four cycles, as shown in Figure 4B. To investigate the reasons for the decrease in activity, XRD and XPS analyses were performed on the catalysts after four cycles. Figure 4C demonstrates that the cycled 2 wt% GQDs/BiOCl-V_O_ had better crystallinity than the pristine 2 wt% GQDs/BiOCl-V_O_. Unexpectedly, the crystallinity of the cycled 2 wt% GQDs/BiOCl-V_O_ might have been transformed to match that of BiOCl. Evidently, high crystallinity indicates fewer defects on the catalyst [37]. According to Figure 4D and Table S3, the relative peak area of oxygen vacancy decreased for the cycled sample, indicating that oxygen vacancy was unstable during the reaction.

### 2.5. Optical and Electrochemical Properties

**Figure 5:**
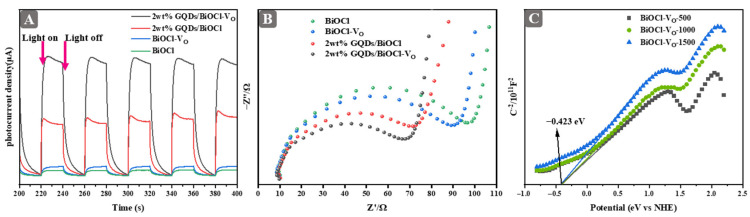
(**A**) Photocurrent response of samples under illumination by visible light. (**B**) Nyquist plot EIS analysis of samples. (**C**) Mott–Schottky curves of BiOCl-V_O_ with 500, 1000, and 1500 frequency.

The separation of photogenerated carriers is essential in photocatalysis. Theoretically speaking, both the electron accumulation and the formation of oxygen vacancies contribute to the migration of electrons, which further enhances the separation efficiency of photogenerated carriers [38,39]. The photocurrent responses of 2 wt% GQDs/BiOCl-V_O_, 2 wt% GQDs/BiOCl, BiOCl-V_O_, and BiOCl samples after deposition on ITO electrodes are displayed in Figure 5A. The order of photocurrent intensities was 2 wt% GQDs/BiOCl-V_O_ > 2 wt% GQDs/BiOCl > BiOCl-V_O_ > BiOCl. This finding elucidated that the integration of oxygen vacancy and GQDs helped enhance the separation of photogenerated carriers [31]. Furthermore, electrochemical impedance spectroscopy (EIS) analysis provided a useful tool for assessing the efficiency of charge pair transfer, as illustrated in Figure 5B. The Nyquist curve arc represents the charge transfer resistance within the material system [40]. The smaller diameter of the EIS Nyquist curve arc indicates a faster separation of photogenerated carriers. Evidently, the arc radius of the 2 wt% GQDs/BiOCl-V_O_ sample is the smallest of all the samples, indicating that this sample has a low impedance with a high carrier separation efficiency. The results reflected in the EIS Nyquist curve are in agreement with the photocurrent. The test further demonstrated that the synergetic effect of oxygen vacancy and GQDs is an efficient approach to enhance the separation of photogenerated carriers of catalysts. Consequently, this method has displayed promise in delivering excellent photocatalytic performance for various applications.

To obtain a clear position for the conduction band, the Mott–Schottky curves of BiOCl-V_O_ were tested using an electrochemical workstation. Using the Nernst equation ($E_{NHE}=E_{Ag/AgCl}+0.127$) [41], the intersection of the slope of the Mott–Schottky curves for both BiOCl-V_O_ samples with the *X*-axis was −0.423 eV in both cases. This value corresponded to the conduction bands (vs. NHE) of BiOCl-V_O_ (Figure 5C). Based on the previous results, it was evident that the introduction of oxygen vacancies led to an alteration in the energy band structure of samples [42].

### 2.6. Active Species Analysis

**Figure 6:**
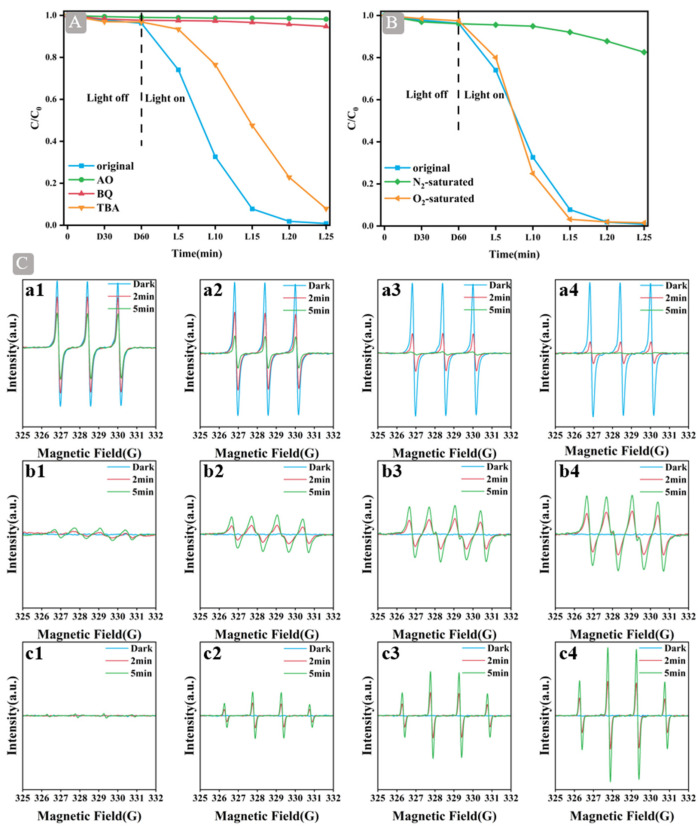
(**A**) Active species capture experiment for RhB degradation on 2 wt% GQDs/BiOCl-V_O_; (**B**) Dissolved oxygen for RhB degradation on 2 wt% GQDs/BiOCl-V_O_; (**C**) ESR signals of TEMPO–h^+^ (**a1**–**a4**), DMPO–·O_2_^−^ (**b1**–**b4**) and DMPO–·OH (**c1**–**c4**) with BiOCl, BiOCl-V_O_, 2 wt% GQDs/BiOCl, and 2 wt% GQDs/BiOCl-V_O_.

The active species of the 2 wt% GQDs/BiOCl-V_O_ were determined using radical trapping experiments and ESR technology. To assess the impact of diverse reactive radicals on the degradation of RhB, trapping experiments were carried out with various scavengers, including ammonium oxalate (AO), *p*-benzoquinone (BQ), and tert-butanol (TBA), to capture h^+^, hydroxyl radicals (·OH), and ·O_2_^−^, respectively (Figure 6A). The trapping experiments showed that h^+^ and ·O_2_^−^ were the main active species and that the ·OH radical played a certain role in the reaction. In an attempt to investigate the degradation of RhB by dissolved oxygen, nitrogen and oxygen were introduced during the degradation process (Figure 6B). The admission of nitrogen diminished the photocatalytic performance; dissolved oxygen played a significant role in the degradation of RhB. Notably, the introduction of dissolved oxygen did not substantially improve the performance. Based on the effect resulting from the loss of oxygen vacancy and the enhancement of the formation of ·O_2_^−^ free radicals, the initial performance was similar to that observed prior to the introduction of dissolved oxygen [43]. The above XPS analysis of 2 wt% GQDs/BiOCl-V_O_ cycled also confirmed this result. Therefore, dissolved oxygen had a considerable influence on the GQDs/BiOCl-V_O_ system.

No ESR signals of DMPO–·OH and DMPO–·O_2_^−^ were detected for any photocatalyst in the dark, but the ESR signals of TEMPO−h^+^ exhibited the strongest intensity (Figure 6C). As shown in Figure 6(Ca1–Ca4), the TEMPO−h^+^ displayed three distinct peaks with an intensity ratio of 1:1:1 under visible-light irradiation. Compared with BiOCl, 2 wt% GQDs/BiOCl-V_O_ exhibited the weakest peak intensity of TEMPO−h^+^ with 5 min of visible-light irradiation. The six characteristic peaks of DMPO–·O_2_^–^ were observed for BiOCl, BiOCl-V_O_, 2 wt% GQDs/BiOCl, and 2 wt% GQDs/BiOCl-V_O_ under visible-light irradiation (Figure 6(Cb1–Cb4)). The intensity of 2 wt% GQDs/BiOCl-V_O_ was higher than that of BiOCl. These results indicate that ·O_2_^−^ radicals are formed by electron reduction [44,45]. As seen in Figure 6(Cc1–Cc4), DMPO–·OH displayed four peaks with an intensity ratio of 1:2:2:1. The highest intensity of DMPO–·OH was observed in 2 wt% GQDs/BiOCl-V_O_. On the basis of ESR technology, the ESR signals of ·O_2_^−^, ·OH, and h^+^ were detected during the light phase, indicating that ·O_2_^–^, ·OH and h^+^ could be generated under light. Additionally, the main active species of 2 wt% GQDs/BiOCl-V_O_ were ·O_2_^−^ and h^+^, and ·OH also played a role in the reaction, corresponding to radical trapping experiments.

The maximum absorption peak of RhB was located at the wavelength of 553 nm. The peak strength decreased with an increase in degradation time. According to the intensity of the absorption peak at 553 nm, RhB degradation activity over 2 wt% GQDs/BiOCl-V_O_ was calculated to be almost 100% at the 20th minute, which hardly changed with the further increase in reaction time (Figure S6C). To provide analysis of the pathways involved in RhB degradation, it is important to understand that the photocatalytic process included two main pathways in the degradation of RhB. First, the degradation process of RhB was facilitated by the removal of the ethyl group. With increasing visible-light irradiation, the absorption peaks of RhB were blue-shifted; that is, shifted from 553 to 501 nm. Moreover, the color of the RhB solution became lighter as duration of irradiation progressed. This shift in the absorption was presumed to be the result of the gradual removal of the ethyl group [46]. Other pathways involved the destruction of the structure of the chromatid ring and the ring-opening decomposition reaction of the benzene ring (Figure S7). The intermediate products after the destruction of the structure of the chromatid, and the products such as oxalic acid generated by the ring-opening of the benzene ring, were then further oxidatively degraded into small molecules under the action of h^+^, ·OH, and ·O_2_^−^. The remaining intermediate product containing C–O–C groups at the end of the reaction was quite stable and difficult to be destroyed by h^+^, ·OH, and ·O_2_^−^, with slow oxidative degradation. Eventually, RhB was mineralized into inorganic salts, water, and carbon dioxide.

### 2.7. Photocatalytic Mechanism

**Figure 7:**
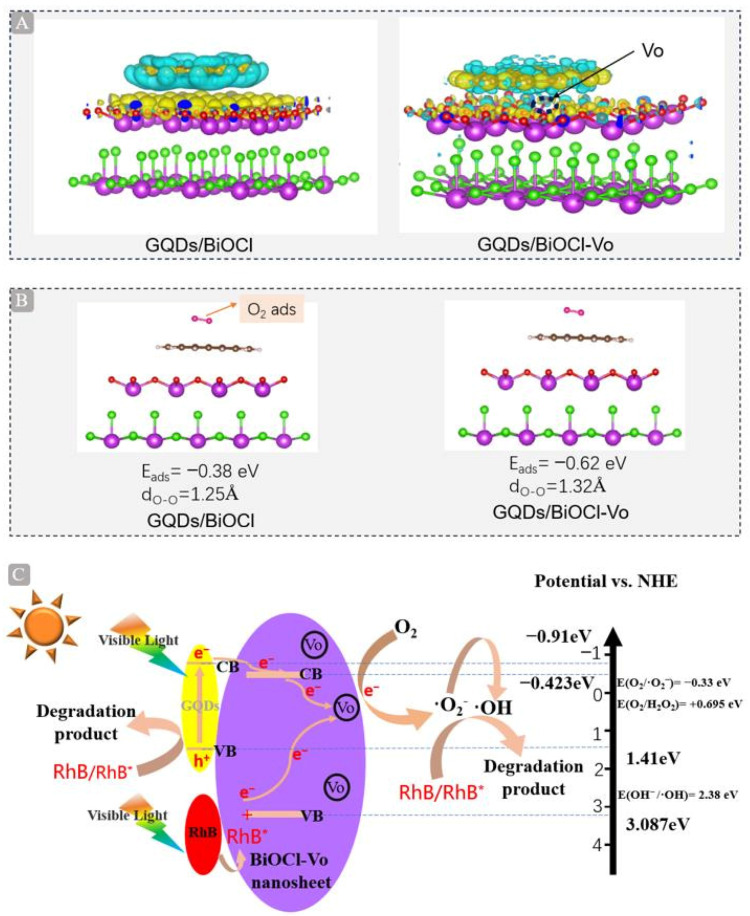
(**A**) Charge density differences of GQDs/BiOCl and GQDs/BiOCl-V_O_ surfaces (the yellow and blue regions represent charge accumulation and consumption). The colored balls represent different atoms: red, oxygen (O); green, chlorine (Cl); purple, bismuth (Bi); gray, carbon (C); white, hydrogen (H). (**B**) Computational models for O_2_ adsorption on GQDs/BiOCl and GQDs/BiOCl-V_O_. (**C**) The proposed degradation mechanism of RhB catalyzed by 2 wt% GQDs/BiOCl-V_O_ under visible light irradiation (420−800 nm).

Differential charge density calculations were conducted to observe the direction of the electron transfer at the interface of the excited GQDs and BiOCl. The H atom of GQDs and the O atom of BiOCl possessed the majority of the electron density, as seen in Figure 7A. Under the synergistic effect of GQDs and oxygen vacancies, strong interactions were generated at the interface. The increase in the yellow region near the oxygen vacancy in BiOCl indicated an increase in charge density, showing that photogenerated electrons migrated to the vicinity of the oxygen vacancy in BiOCl to enhance the adsorption of oxygen molecules. We speculate that GQDs may act as photosensitizers to generate photogenerated electrons that will transfer to adjacent positions of oxygen vacancies in BiOCl (as indicated by the arrow). These results provide a theoretical basis for charge transfer and capture between GQDs and BiOCl-V_O_. In addition, the adsorption energy of O_2_ for the GQDs/BiOCl and GQDs/BiOCl-V_O_ models was investigated (Figure 7B). The results indicated that O_2_ is more strongly adsorbed on GQDs/BiOCl-V_O_ (E_ads_(O_2_) = −0.62 eV) than on GQDs/BiOCl (E_ads_(O_2_) = −0.38 eV), with the O−O bond length stretching from 1.25 Å to 1.32 Å. In summary, the formation of GQDs and oxygen vacancies promotes the adsorption and activation of oxygen molecules, and effectively promotes the separation and transport of photogenerated charges, thereby enhancing the performance of photocatalytic degradation of RhB. 

Based on the above experiments and DFT calculation results, a preliminary mechanism for the photocatalytic degradation of RhB on GQDs/BiOCl-V_O_ has been proposed (Figure 7C). The calculations involved determining the conduction band (CB) and valence band (VB) edges of the semiconductor using the following equation:

(1) $$E_{g}=E_{VB}-E_{CB}$$

where $E_{g}$ is the band gap energy, and $E_{CB}$ and $E_{VB}$ are the CB and VB edge potentials of the photocatalysis, respectively. The band gap of BiOCl-V_O_ was 3.51 eV, as verified by DRS; conversely, BiOCl cannot absorb visible light. Moreover, E_CB_, obtained from the Mott−Schottky curve, was −0.423 eV (vs. NHE). The E_VB_ of BiOCl-V_O_ was determined as 3.087 eV (vs. the NHE) by calculation following Equation (1). The E_CB_ and E_VB_ of GQDs are, respectively, −0.91 eV and 1.41 eV, sourced from reports published by other researchers [47]. When visible light was absorbed, RhB formed RhB^*^ and e^−^, and GQDs created h^+^ and e^−^ (Equation (2)). The e^−^ of GQDs and RhB transforms the CB of BiOCl-V_O_ following Equation (3). The CB edge potential of BiOCl-V_O_ was more negative than the O_2_/O_2_^−^ potential (−0.33 eV vs. NHE) and the O_2_/H_2_O_2_ potential (+0.695 eV vs. NHE); therefore, the electrons reduced O_2_ to ·O_2_^−^ (Equation (4)) and ·O_2_^−^ transformed ·OH (Equations (5)−(7)). Eventually, RhB was degraded by h^+^, ·O_2_^−^, and ·OH following Equation (8). The possible reactions are described as follows:

GQDs + hν → e^−^, h^+^ (GQDs) and RhB + hν → RhB* + e^−^ (RhB) (2)

e^−^ (GQDs)/(RhB) → e^−^ (BiOCl-V_O_) (3)

e^−^ (BiOCl-V_O_) + O_2_ → ·O_2_^−^ (4)

·O_2_^−^ + H^+^ →HOO (5)

HOO· + e^−^ + H^+^ → H_2_O_2_ (6)

H_2_O_2_ + e^−^ → ·OH + OH^−^ (7)

GQDs + hν → e^−^, h^+^ (GQDs) and RhB + hν → RhB* + e^−^ (RhB) (8)

## 3. Materials and Methods

### 3.1. Materials

Bismuth (III) nitrate pentahydrate (Bi(NO_3_)_3_·5H_2_O), D-Mannitol (C_6_H_14_O_6_), and sodium chloride were purchased from Sinopharm Chemical Reagent Co., Ltd. (Shanghai, China). Polyvinylpyrrolidone (PVP, average Mw ~55,000) was obtained from Sigma-Aldrich Co., Ltd. (St. Louis, MO, USA). GQDs (1 mg/mL) were bought from Aladdin Co., Ltd. (Shanghai, China). All reagents were analytically pure and commercially available, and no further purification was carried out.

### 3.2. Preparation of Photocatalysts

BiOCl-V_O_ was synthesized using a refined method introduced by Zhang et al. [48]. In this method, 1 mmol of Bi(NO_3_)_3_·5H_2_O and 0.400 g of PVP were dissolved in 30 mL of 0.1 mol/L mannitol solution. After 10 min of stirring, a saturated NaCl solution (5 mL) was gradually added dropwise into the above-mentioned solution. Subsequently, 1 mg/mL GQDs solution (1wt%, 2 wt%, and 3wt%) was added, with continuous stirring for 10 min. The suspension was transferred into a Teflon-lined stainless steel autoclave of 100 mL capacity and maintained at 160 °C for 3 h. The final products were subject to repeated alternation of washing and centrifugation using deionized water and absolute ethanol and then dried at 60 °C for 8 h in air. The obtained samples were denoted as 1wt% GQDs/BiOCl-V_O_, 2 wt% GQDs/BiOCl-V_O_, and 3 wt% GQDs/BiOCl-V_O_. Meanwhile, pure BiOCl-V_O_ and GQDs/BiOCl samples were synthesized using the same procedure as that used for the synthesis of the GQDs/BiOCl-V_O_ sample, except without the addition of GQDs and PVP, respectively. The obtained samples were denoted as 1 wt% GQDs/BiOCl, 2 wt% GQDs/BiOCl, and 3 wt% GQDs/BiOCl.

### 3.3. Characterization

The crystalline structure of the synthesized photocatalysts was characterized via XRD (XRD-7000, SHIMADZU, Kyoto, Japan), equipped with Ni-filtered Cu Kα radiation. TEM and HRTEM images were collected using a FEI Tecnai G2 F20 S-TWIN instrument (Hillsboro, OR, USA) operating at an accelerating voltage of 200 kV. The DRS were determined on a Varian Cary 100 spectrometer (Varian Co., Palo Alto, CA, USA), utilizing large integrating sphere accessories and employing standard BaSO_4_ serving as a reference. The surface composition and state of elements were recognized using XPS (Thermo Fisher Scientific K-Alpha, Waltham, MA, USA). The ESR signals were collected by a JEOL JES-FA200 spectrometer (Akishima, Japan), and the excitation source was a 500 W Xe lamp equipped with an IR-cutoff filter (λ < 800 nm) and a UV-cutoff filter (λ > 420 nm). Test conditions were as follows: room temperature, central magnetic field 3512 G, scanning width 100.0 G, and microwave frequency 9.5 GHz. The active species (including h^+^, ·O_2_^−^, and ·OH) generated on the surfaces of samples were trapped using 5, 5-dimethyl-1-pyrroline-N-oxide (DMPO methanol solution) or 2,2,6,6-Tetramethylpiperidoxyl (TEMPO water solution) as a spin trap. The powder samples were evenly dispersed in the DMPO or TEMPO solution. After undergoing oscillation, a trace solution was carefully collected using a capillary quartz tube for further analysis. Photos were taken using a digital camera (Canon 70D). Nitrogen sorption experiments were conducted on a Micromeritics ASAP 2460 M (Micromeritics Instrument Corporation, Atlanta, GA, USA), as well as on a Tristar system apparatus, both at −196 °C. The micropore volume and specific surface area were calculated using the t-plot and BET methods, respectively. FTIR spectra were recorded on an FTIR spectrometer (BRUKER TENSOR 27, Burladingen, Germany).

### 3.4. Photoeletrochemical Measurements

The photoelectrochemical experiment was measured using a CHI-660E electrochemical workstation (ArtisanTG, Champaign, IL, USA). The different photocatalysts were deposited on a 5 mm × 5 mm ITO conductive glass that served as the working electrode. A Pt plate was used as the counter electrode and an Ag/AgCl electrode was used as the reference electrode. All electrodes were immersed in 0.5 M Na_2_SO_4_ electrolyte (30 mL) at 25 °C. Before the photocurrent measurement, N_2_ gas was purged into the Na_2_SO_4_ aqueous solution to remove dissolved oxygen for 30 min. The light source was a 500 W Xe-arc lamp equipped with an IR cutoff filter (λ < 800 nm) and a UV cutoff filter (λ > 420 nm). The Mott-Schottky curves were obtained at the selected frequencies (500, 1000, and 1500 Hz). After a 30 min delay in darkness, EIS was measured at frequencies ranging from 1000 Hz to 1 MHz, with a DC potential of 0.2 V applied to the open circuit potential.

### 3.5. Photocatalytic Test

Photocatalytic properties of different samples were evaluated using RhB (40 mg/L) and norfloxacin (20 mg/L) as the target pollutants. The light source was a 500 W xenon lamp equipped with IR and UV cutoff filters, so as to obtain an illumination range of 420−800 nm. The photocatalytic activity under UV light irradiation used a Hg lamp as the light source. In the process, 0.04 g of photocatalyst was added to 80 mL of RhB solution, and evenly dispersed in the solution through 1 h of stirring, reaching a dark adsorption-desorption equilibrium. Then, the light source was turned on, and sampling was performed once every five minutes until the reaction time was up to 25 min. After centrifugation, the supernatant was taken and measured for absorbance at 553 nm using a Varian Cary 100 spectrophotometer. The RhB removal rate was calculated based on the degradation percentage of C/C_0_, where C is the maximum intensity of RhB absorption spectra for each irradiated time interval at a wavelength of 553 nm, and C_0_ is the absorption intensity of the initial RhB concentration.

### 3.6. DFT Calculations

All DFT calculations were performed using Dmol3 in Materials Studio. The Perdew-Burke-Ernzerhof (PBE) exchange-correlation functional based on the General Gradient Approximation was used to describe the interaction between electrons. Atomic orbitals were expanded using a double numerical plus polarization (DNP) basis set. The convergence criteria for energy, force, and displacement were set to 10^−5^ Hartree, 0.002 Hartree/Å, and 0.005 Å, respectively. A K-points grid with density of 3 × 3 × 1 and 7 × 7 × 1 was selected for numerical integration in Brillouin zone. We used a 4 × 4 supercell to model BiOCl (001) surface and a vacuum region of 18 Å was used to avoid interactions between two adjacent periodic supercells along the Z direction. The average adsorption of O_2_ was defined as:

$$E_{ad}=E_{O_{2}/surface}-E_{surface}-E_{O_{2}}$$

where $E_{O_{2}/surface}$ is the total energy of the adsorbed system, $E_{surface}$ is the energy of the surface, and $E_{O_{2}}$ is the energy of $O_{2}$.

## 4. Conclusions

In summary, BiOCl nanosheets modified with GQDs and oxygen vacancy were successfully synthesized via a simple hydrothermal method. The introduction of GQDs and oxygen vacancies had a significant influence on the photodegradation efficiency. The 2 wt% GQDs/BiOCl-V_O_ exhibited the highest photocatalytic degradation for RhB. Such improved photocatalytic activity was ascribed to the synergistic effects of GQDs and oxygen vacancy, leading to enhanced light harvesting and effective separation of photogenerated charges in the heterophase GQDs/BiOCl-V_O_ system, as evidenced by the experimental analysis and DFT calculations. In addition, the potential mechanism of organic pollutant degradation was discussed. Based on the experimental results of radical trapping experiments and ESR, h^+^ and ·O_2_^−^ were identified as the species involved in the photocatalytic degradation of RhB. This work delves into the important role of graphene quantum dots and oxygen vacancies in the interface structure and photocatalytic performance of BiOCl and provides a new strategy for designing catalysts with efficient active sites through the interactive coupling effect of non-precise metals and oxygen vacancy. In ongoing explorations, we will focus on improving preparation methods and optimizing heterostructures to enhance the cycling stability of catalysts and their practical application efficiency, thereby expanding the industrial application range of catalysts.

## Data Availability

Data are contained within the article and Supplementary Materials.

## Supplementary Materials

The following supporting information can be downloaded at: https://www.mdpi.com/article/10.3390/molecules29061362/s1, Scheme S1: Schematic diagram of the GQDs/BiOCl-V_O_ preparation process; Figure S1: (A) XRD patterns of different content GQDs/BiOCl-VO; (B) The enlarged XRD patterns of different samples; (C) The appearance of the material; (D) Plot of (αhν)1/2 against hν for BiOCl and BiOCl-VO; Figure S2: HOMO and LUMO of GQDs/BiOCl and GQDs/BiOCl-VO. Note: the colored balls represent different atoms: red, oxygen (O); green, chlorine (Cl); purple, bismuth (Bi); gray, carbon (C); white, hydrogen (H); Figure S3: (A) HRTEM of the GQDs; (B) TEM of the GQDs/BiOCl-VO; Figure S4: (A) Nitrogen adsorption-desorption isotherms of as-prepared samples; (B) The XPS full spectrum; Figure S5: Degradation curves of RhB over the BiOCl-VO sample prepared at 160℃ for holding different time (A), prepared at 3 h with different temperature (B); The RhB degradation ratio of GQDs/BiOCl system (C) and GQDs/BiOCl-VO system (D) under visible-light irradiation; Figure S6: (A) The degradation performance of RhB under UV light irradiation; (B) The degradation performance of norfloxacin under visible-light irradiation; (B) UV-vis spectrograms of degradation of RhB; Figure S7: The sketch of RhB degradation process. Table S1: The calculation of crystal size from XRD results; Table S2: BET surface area and average pore diameter for the catalysts; Table S3: XPS relative peak area percentage of Cl 2p, Bi 4f, C 1s and O 1s.

## References

[1] He M., Xu Z., Hou D., Gao B., Cao X., Ok Y.S., Rinklebe J., Bolan N.S., Tsang D.C.W. (2022). Waste-derived biochar for water pollution control and sustainable development. Nat. Rev. Earth Environ..

[2] Guo Y., Tong X., Yang N. (2023). Photocatalytic and electrocatalytic generation of hydrogen peroxide: Principles, catalyst design and performance. Nano-Micro Lett..

[3] Du Z., Gong K., Yu Z., Yang Y., Wang P., Zheng X., Wang Z., Zhang S., Chen S., Meng S. (2023). Photoredox coupling of CO_2_ reduction with benzyl alcohol oxidation over ternary metal chalcogenides (Zn_m_In_2_S_3+m_, m = 1 − 5) with regulable products selectivity. Molecules.

[4] Meng S., Chen C., Gu X., Wu H., Meng Q., Zhang J., Chen S., Fu X., Liu D., Lei W. (2021). Efficient photocatalytic H_2_ evolution, CO_2_ reduction and N_2_ fixation coupled with organic synthesis by cocatalyst and vacancies engineering. Appl. Catal. B Environ..

[5] Wang G., Xiong S., Chen Y., Wang C., Lv S., Jia K., Xiang Y., Liu J., Liu C., Li Z. (2023). Internal magnetic-field-enhanced photogenerated charge separation in ferromagnetic TiO_2_ surface heterojunctions. J. Mater. Sci. Technol..

[6] Mengting Z., Kurniawan T.A., Duan L., Song Y., Hermanowicz S.W., Othman M.H.D. (2022). Advances in BiOX-based ternary photocatalysts for water technology and energy storage applications: Research trends, challenges, solutions, and ways forward. Rev. Environ. Sci. Biotechnol..

[7] Li W., Mao Y., Liu Z., Zhang J., Luo J., Zhang L., Qiao Z.-A. (2023). Chelated ion-exchange strategy toward BiOCl mesoporous single-crystalline nanosheets for boosting photocatalytic selective aromatic alcohols oxidation. Adv. Mater..

[8] Wenbin J., Hongyi L., Beverly Qian Ling L., Houjuan Z., Jingxiang L., Jerry Zhi Xiong H., Karen Yuanting T., Zibiao L., Xian Jun L., Enyi Y. (2023). Role of oxygen vacancy in metal oxides for photocatalytic CO_2_ reduction. Appl. Catal. B Environ. Energy.

[9] Zhang L., Wang W., Jiang D., Gao E., Sun S. (2015). Photoreduction of CO_2_ on BiOCl nanoplates with the assistance of photoinduced oxygen vacancies. Nano Res..

[10] Cui D., Wang L., Xu K., Ren L., Wang L., Yu Y., Du Y., Hao W. (2018). Band-gap engineering of BiOCl with oxygen vacancies for efficient photooxidation properties under visible-light irradiation. J. Mater. Chem. A.

[11] Zhang C., Deng Y., Wan Q., Zeng H., Wang H., Yu H., Huang J. (2024). Built-in electric field boosted exciton dissociation in sulfur doped BiOCl with abundant oxygen vacancies for transforming the pathway of molecular oxygen activation. Appl. Catal. B Environ. Energy.

[12] Qin H., Sun J. (2022). Xia, D. Xu, H. Yu, Q. Zheng, Y.; Shi, Y. Boosting nonradical process in BiOI/BiOCl heterostructure by interface oxygen vacancies. Chem. Eng. J..

[13] Yang Z., Shi Y., Li H., Mao C., Wang X., Liu X., Liu X., Zhang L. (2022). Oxygen and chlorine dual vacancies enable photocatalytic O_2_ dissociation into monatomic reactive oxygen on BiOCl for refractory aromatic pollutant removal. Environ. Sci. Technol..

[14] Ren Q., He Y. (2022). Wang, H. Sun, Y.; Dong, F., Photo-switchable oxygen vacancy as the dynamic active site in the photocatalytic NO oxidation reaction. ACS Catal..

[15] Fan M., Wang Z., Sun K., Wang A., Zhao Y., Yuan Q., Wang R., Raj J., Wu J., Jiang J. (2023). N-B-OH site-activated graphene quantum dots for boosting electrochemical hydrogen peroxide production. Adv. Mater..

[16] Yan Y., Gong J., Chen J., Zeng Z., Huang W., Pu K., Liu J., Chen P. (2019). Recent advances on graphene quantum dots: From chemistry and physics to applications. Adv. Mater..

[17] Chen P., Liu H., Sun Y., Li J., Cui W., Zhang W., Yuan X., Wang Z., Zhang Y., Dong F. (2020). Bi metal prevents the deactivation of oxygen vacancies in Bi_2_O_2_CO_3_ for stable and efficient photocatalytic NO abatement. Appl. Catal. B Environ..

[18] Yu Q., Wang X., Wu W., Feng X., Kong D., Khan U., Ren X., Li L. (2023). In situ encapsulation of graphene quantum dots in highly stable porphyrin metal-organic frameworks for efficient photocatalytic CO_2_ reduction. Molecules.

[19] Jia D., Li X., Chi Q., Low J., Deng P., Wu W., Wang Y., Zhu K., Li W., Xu M. (2022). Direct electron transfer from upconversion graphene quantum dots to TiO_2_ enabling infrared light-driven overall water splitting. Research.

[20] Mandal S., Adhikari S., Choi S., Lee Y., Kim D.H. (2022). Fabrication of a novel Z-scheme Bi_2_MoO_6_/GQDs/MoS_2_ hierarchical nanocomposite for the photo-oxidation of ofloxacin and photoreduction of Cr(VI) as aqueous pollutants. Chem. Eng. J..

[21] Sahu R.S., Dubey A., Shih Y.h. (2022). Novel metal-free in-plane functionalized graphitic carbon nitride with graphene quantum dots for effective photodegradation of 4-bromophenol. Carbon.

[22] Zhang Y., Miao N., Xin X., Wang Y., Zhu J., Guo P., Li X. (2022). Boosting the photocatalytic performance via defect-dependent interfacial interactions from electrostatic adsorption to chemical bridging. Nano Energy.

[23] Zou Y., Weng J., Qin Z., Zhang Y., Ji S., Zhang H. (2023). Metal-organic framework and graphene quantum dot-incorporated nanofibers as dual stimuli-responsive platforms for day/night antibacterial bio-protection. Chem. Eng. J..

[24] Cui Y., Wang T., Liu J., Hu L., Nie Q., Tan Z., Yu H. (2021). Enhanced solar photocatalytic degradation of nitric oxide using graphene quantum dots/bismuth tungstate composite catalysts. Chem. Eng. J.

[25] Zhang K., Liu C., Huang F., Zheng C., Wang W. (2006). Study of the electronic structure and photocatalytic activity of the BiOCl photocatalyst. Appl. Catal. B Environ. Energy.

[26] Guan M., Xiao C., Zhang J., Fan S., An R., Cheng Q., Xie J., Zhou M., Ye B., Xie Y. (2013). Vacancy associates promoting solar-driven photocatalytic activity of ultrathin bismuth oxychloride nanosheets. J. Am. Chem. Soc..

[27] Ren Y., Zou J., Jing K., Liu Y., Guo B., Song Y., Yu Y., Wu L. (2019). Photocatalytic synthesis of N-benzyleneamine from benzylamine on ultrathin BiOCl nanosheets under visible light. J. Catal..

[28] Quan B., Liu W., Liu Y., Zheng Y., Yang G., Ji G. (2016). Quasi-noble-metal graphene quantum dots deposited stannic oxide with oxygen vacancies: Synthesis and enhanced photocatalytic properties. J. Colloid Interface Sci..

[29] Xiong S., Bao S., Wang W., Hao J., Mao Y., Liu P., Ouyang D. (2020). Understanding the effects of co-exposed facets on photocatalytic activities and fuel desulfurization performance in BiOCl singlet-crystalline sheets. J. Hazard. Mater..

[30] Wang C., Li S., Cai M., Yan R., Dong K., Zhang J., Liu Y. (2022). Rationally designed tetra (4-carboxyphenyl) porphyrin/graphene quantum dots/bismuth molybdate Z-scheme heterojunction for tetracycline degradation and Cr (VI) reduction: Performance, mechanism, intermediate toxicity appraisement. J. Colloid Interface Sci..

[31] Xiong S., Bao S., Wang W., Hao J., Mao Y., Liu P., Huang Y., Duan Z., Lv Y., Ouyang D. (2022). Surface oxygen vacancy and graphene quantum dots co-modified Bi_2_WO_6_ toward highly efficient photocatalytic reduction of CO_2_. Appl. Catal. B Environ..

[32] Xu K., Xu D., Li Z., Zhang S., Tong L., Peng J., Zhang S., Shen J., Chen X. (2022). Enhanced visible-light photocatalytic degradation of ciprofloxacin hydrochloride by bulk iodine doped BiOCl with rich oxygen vacancy. Appl. Surf. Sci..

[33] Zhao X., Deng B., Li F., Huang M., Sun Y., Li J., Dong F. (2021). Efficient photocatalytic toluene degradation over heterojunction of GQDs@BiOCl ultrathin nanosheets with selective benzoic acid activation. J. Hazard. Mater..

[34] Zhang P., Qiu Y., Yang S., Jiao Y., Ji C., Li Y., Chen B., Fan H. (2017). Oxygen-deficient bismuth oxychloride nanosheets: Superior photocatalytic performance. Mater. Res. Bull..

[35] Li X., Dong Q., Li F., Zhu Q., Tian Q., Tian L., Zhu Y., Pan B., Padervand M., Wang C. (2024). Defective Bi@BiOBr/C microrods derived from Bi-MOF for efficient photocatalytic NO abatement: Directional regulation of interfacial charge transfer via carbon–loading. Appl. Catal. B Environ..

[36] Wu X., Oropeza F.E., den Boer D., Kleinschmidt P., Hannappel T., Hetterscheid D.G.H., Hensen E.J.M., Hofmann J.P. (2023). Thermally induced oxygen vacancies in BiOCl nanosheets and their impact on photoelectrochemical performance. ChemPhotoChem..

[37] Zheng Y., Fu K., Yu Z., Su Y., Han R., Liu Q. (2022). Oxygen vacancies in a catalyst for VOCs oxidation: Synthesis, characterization, and catalytic effects. J. Mater. Chem. A.

[38] Yang J., Miao H., Jing J., Zhu Y., Choi W. (2021). Photocatalytic activity enhancement of PDI supermolecular via π-π action and energy level adjusting with graphene quantum dots. Appl. Catal. B Environ..

[39] Lu Y., Chen M., Jiang L., Cao J.-j., Li H., Lee S.C., Huang Y. (2022). Oxygen vacancy engineering of photocatalytic nanomaterials for enrichment, activation, and efficient removal of nitrogen oxides with high selectivity: A review. Environ. Chem. Lett..

[40] Iqbal W., Yang B., Zhao X., Rauf M., Mohamed I.M., Zhang J., Mao Y. (2020). Facile one-pot synthesis of mesoporous g-C_3_N_4_ nanosheets with simultaneous iodine doping and N-vacancies for efficient visible-light-driven H_2_ evolution performance. Catal. Sci. Technol..

[41] Osorio S.C., Biesheuvel P.M., Spruijt E., Dykstra J.E., van der Wal A. (2022). Modeling micropollutant removal by nanofiltration and reverse osmosis membranes: Considerations and challenges. Water Res..

[42] Li F., Liu G., Liu F., Wu J., Yang S. (2023). Synergetic effect of CQD and oxygen vacancy to TiO_2_ photocatalyst for boosting visible photocatalytic NO removal. J. Hazard. Mater..

[43] Chen F., Ma Z., Ye L., Ma T., Zhang T., Zhang Y., Huang H. (2020). Macroscopic spontaneous polarization and surface oxygen vacancies collaboratively boosting CO_2_ photoreduction on BiOIO_3_ single crystals. Adv. Mater..

[44] Hu X., Wang J., Wang J., Deng Y., Zhang H., Xu T., Wang W. (2022). β particles induced directional inward migration of oxygen vacancies: Surface oxygen vacancies and interface oxygen vacancies synergistically activate PMS. Appl. Catal. B Environ..

[45] Yang M., He L., Shi Z., Mei J., Liu C., Yang B., Sun S. (2023). An Unprecedented Strategy to Fabricate Inside/Surface Homojunction in Bismuth Oxychloride for Efficient Photocatalysis. J. Phys. Chem. C..

[46] Fu H., Zhang S., Xu T., Zhu Y., Chen J. (2008). Photocatalytic degradation of RhB by fluorinated Bi_2_WO_6_ and distributions of the intermediate products. Environ. Sci. Technol..

[47] Ebrahimi M., Samadi M., Yousefzadeh S., Soltani M., Rahimi A., Chou T.-c., Chen L.-C., Chen K.-H., Moshfegh A.Z. (2017). Improved solar-driven photocatalytic activity of hybrid graphene quantum dots/ZnO nanowires: A direct Z-scheme mechanism. ACS Sustain. Chem. Eng..

[48] Zhang Y., Xu Z., Wang Q., Hao W., Zhai X., Fei X., Bi Y. (2021). Unveiling the activity origin of ultrathin BiOCl nanosheets for photocatalytic CO_2_ reduction. Appl. Catal. B Environ..

