# Supplementary material for: Boosting Visible-Light Photocatalytic Activity of BiOCl Nanosheets via Synergetic Effect of Oxygen Vacancy Engineering and Graphene Quantum Dots-Sensitization
Source: Molecules. 2024 Mar 19;29(6):1362. doi: 10.3390/molecules29061362 (PMC10975013; doi:10.3390/molecules29061362)
Supplement: Supplementary file 1 [file molecules-29-01362-s001.zip › molecules-2884829-supplementary.pdf]

molecules

Supplementary Materials

Boosting Visible-Light Photocatalytic Activity of BiOCl Nanosheets via Synergetic Effect of Oxygen Vacancy Engineering and Graphene Quantum Dots-Sensitization

Zisheng Shi [1], Wei Chen [2,*], Yin Hu [2,*], Fen Zhang [2], Lingling Wang [2], Dan Zhou [1], Xuanye Chen [2] and Sugang Meng [3]

[1] School of Environment and Chemical Engineering, Nanchang Hangkong University, Nanchang 330063, China; victoryoflion@163.com (Z.S.); zhoudan@nchu.edu.cn (D.Z.)
[2] Research Institute of Applied Chemistry, Jiangxi Academy of Sciences, Nanchang 330096, China; cathyzf@163.com (F.Z.); sunnyskywang@163.com (L.W.); xuanyechen11@163.com (X.C.)
[3] Key Laboratory of Green and Precise Synthetic Chemistry and Applications, Ministry of Education, Huaibei Normal University, Huaibei 235000, China; mengsugang@126.com
* Correspondence: chenwei@jxas.ac.cn (W.C.); huyin@jxas.ac.cn (Y.H.); Tel.: +86-13647910692 (W.C.)

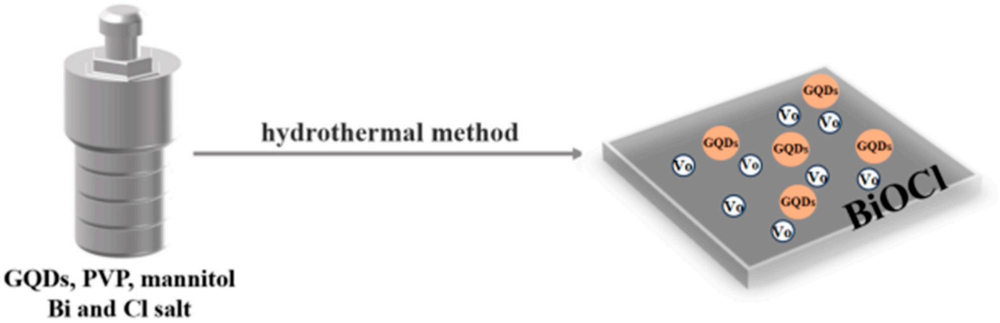

Scheme S1. Schematic diagram of the GQDs/BiOCl-V_O preparation process.

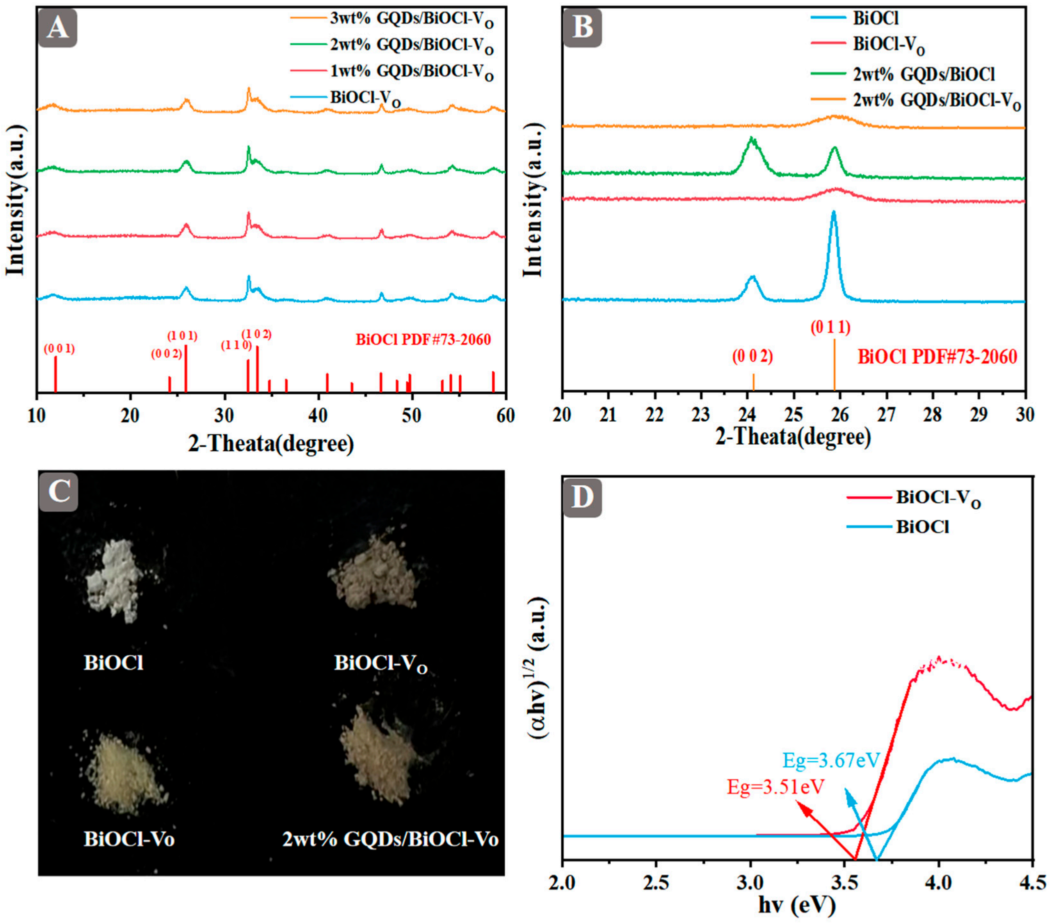

Figure S1. (A) XRD patterns of different content GQDs/BiOCl-V_O; (B) The enlarged XRD patterns of different samples; (C) The appearance of the material; (D) Plot of $(\alpha h\nu)^{1/2}$ against hν for BiOCl and BiOCl-V_O.

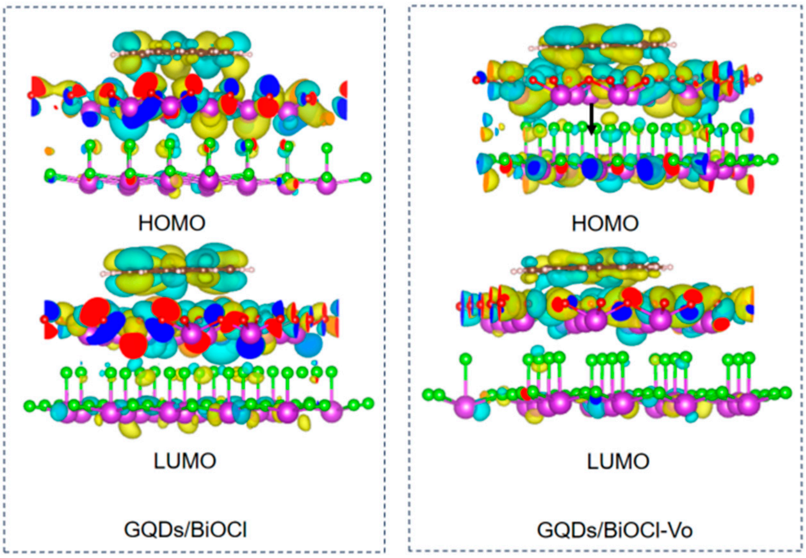

Figure S2. HOMO and LUMO of GQDs/BiOCl and GQDs/BiOCl-V_O. Note: the colored balls represent different atoms: red, oxygen (O); green, chlorine (Cl); purple, bismuth (Bi); gray, carbon (C); white, hydrogen (H).

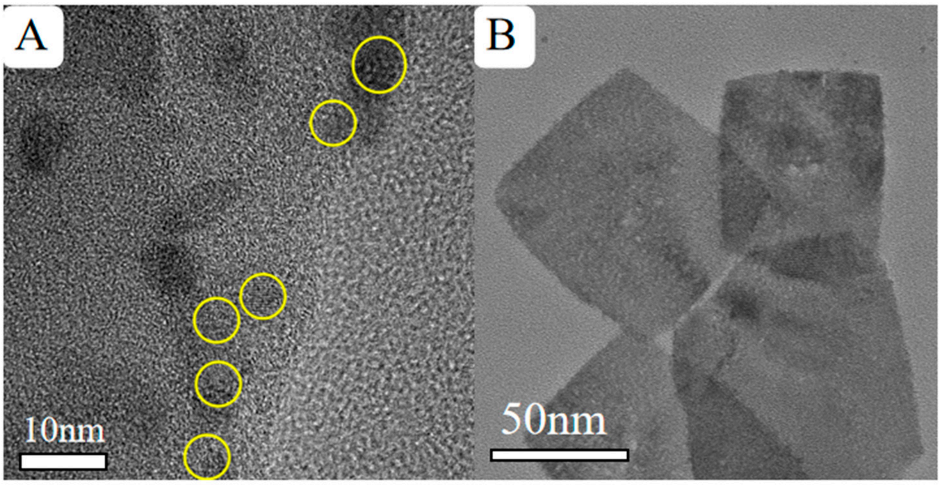

Figure S3. (A) HRTEM of the GQDs; (B) TEM of the GQDs/BiOCl-VO.

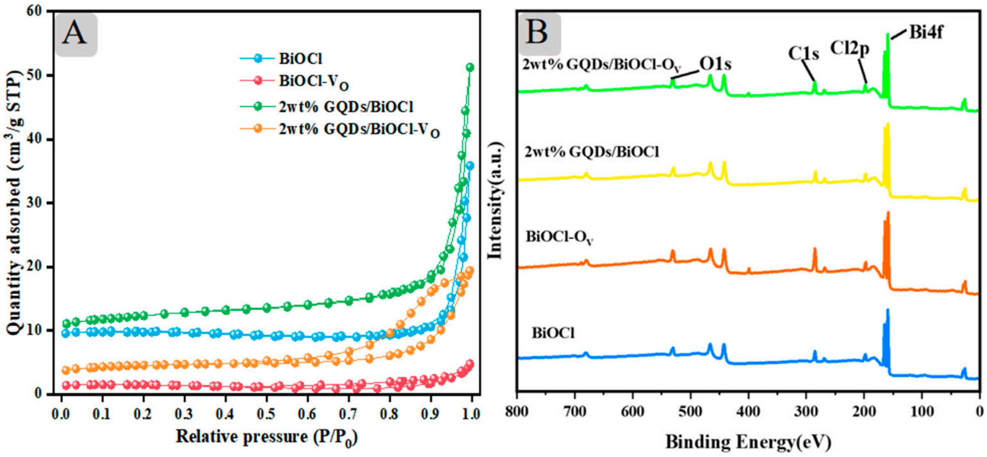

Figure S4. (A) Nitrogen adsorption-desorption isotherms of as-prepared samples; (B) The XPS full spectrum.

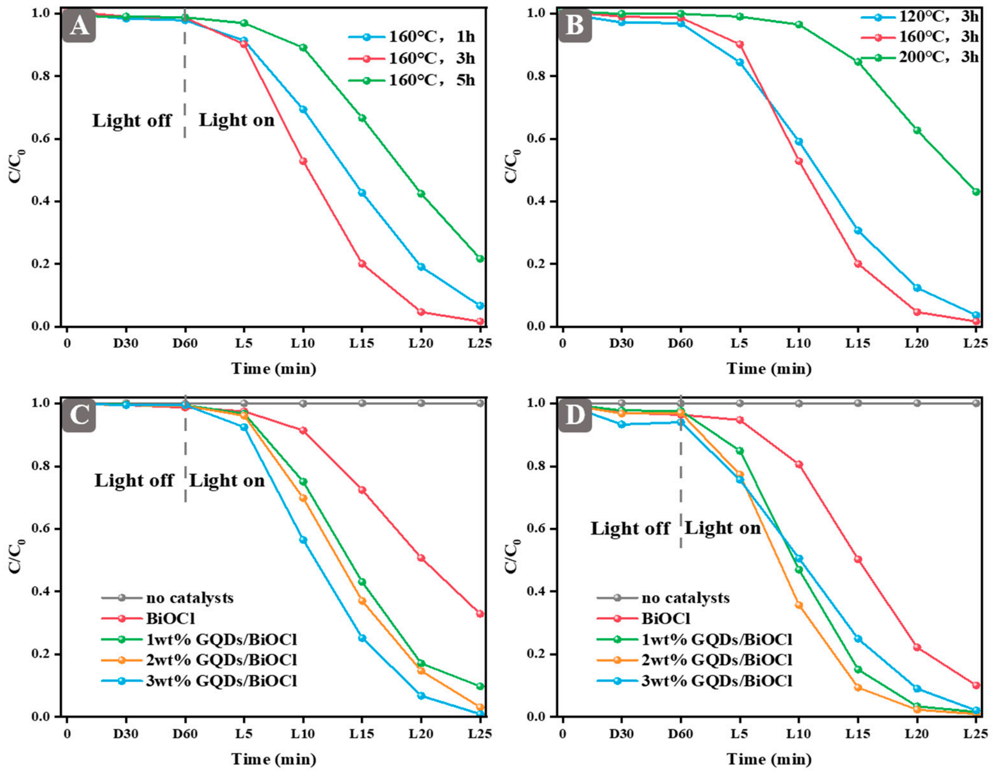

Figure S5. Degradation curves of RhB over the BiOCl-Vo sample prepared at 160℃ for holding different time (A), prepared at 3 h with different temperature (B); The RhB degradation ratio of GQDs/BiOCl system (C) and GQDs/BiOCl-Vo system (D) under visible-light irradiation.

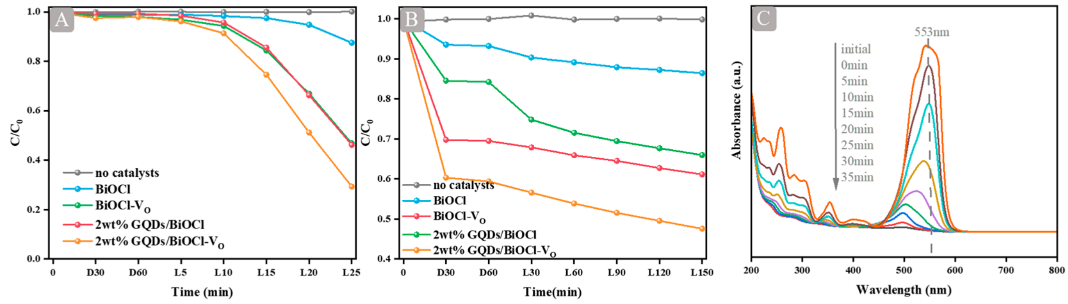

Figure S6. (A) The degradation performance of RhB under UV light irradiation; (B) The degradation performance of norfloxacin under visible-light irradiation; (B) UV–vis spectrograms of degradation of RhB.

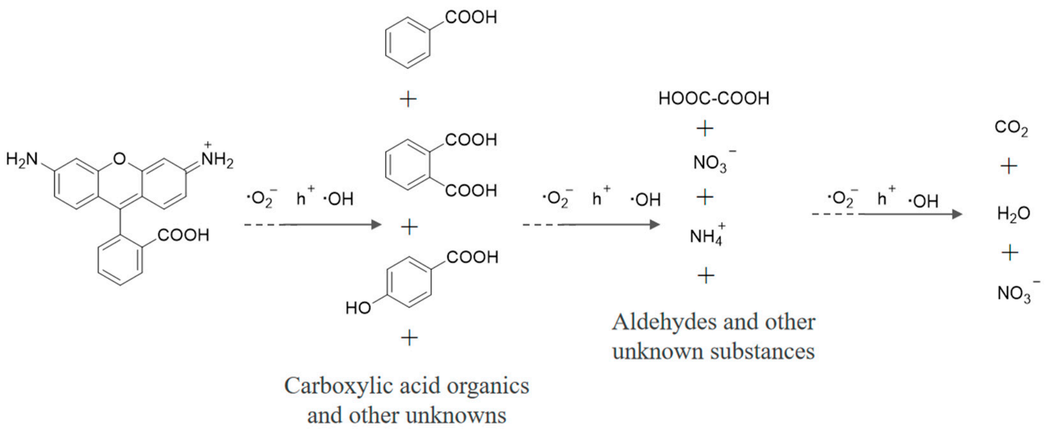

Figure S7. The sketch of RhB degradation process.

Table S1. Microstructural feature of as-prepared samples obtained from XRD analysis.

Catalyst	a (Å)	b (Å)	c (Å)	α= β = γ （ ° ）	Crystallite size (nm)	Cell volume (Å³)
BiOCl	3.895	3.895	7.376	90	30.9	111.75
BiOCl-V_O	3.865	3.865	7.499	90	18.0	110.94
2wt% GQDs/BiOCl	3.859	3.859	7.370	90	18.9	111.66
2wt% GQDs/BiOCl-V_O	3.895	3.895	7.266	90	13.4	110.98

Table S2. BET surface area and average pore diameter for the catalysts.

Catalyst	BiOCl	BiOCl-V_O	2wt% GQDs/BiOCl	2wt% GQDs/BiOCl-V_O
BET surface area (m^2/g)	5.20	1.25	12.10	5.58
Average pore dimeter (nm)	32.97	18.36	21.39	18.16

Table S3. XPS relative peak areas percentage of Cl 2p, Bi 4f, C 1s and O 1s.

Element	Relative Peak areas (%) / Sample	BiOCl	BiOCl-Vo	2wt% GQDs/BiOCl	2wt% GQDs/BiOCl-Vo	2wt% GQDs/BiOCl-Vo cycled
**Cl 2p**	Cl $2p_{1/2}$	33.8	35.6	34.1	34.3	
	Cl $2p_{3/2}$	66.2	64.4	65.9	65.7	
**Bi 4f**	Bi $4f_{7/2}$	43.3	42.8	42.7	43.0	
	Bi $4f_{5/2}$	56.7	57.2	57.3	57.0	
**C 1s**	C–C	36.6	52.1	51.2	55.5	
	C–O–C	63.4	33.3	30.8	33.5	
	O–C=O		14.6	18.0	11.0	
**O 1s**	Crystal lattice	25.6	17.7	9.8	10.0	17.5
	Oxygen vacancies	21.1	33.6	39.9	61.2	33.0
	Surface absorbed oxygen	53.3	48.7	50.3	28.8	49.5
